# Extra-uterine Pregnancy, with Report of a Case of Simultaneous Pregnancy in Both Tubes

**Published:** 1901-04

**Authors:** C. R. Robins

**Affiliations:** Richmond, Va., Instructor in Abdominal Surgery and Gynecology, and Visiting Obstetrician, Medical College of Virginia


ATLANTA

### Journal-Record of Medicine.

Successor to Atlanta Medical and Surgical Journal, Established 1855, and Southern Medical Record, Established 1870.

VOL. III. APRIL, 1901. No. 1.

BERNARD WOLFF, M.D., EDITOR, Nos. 319–20 Prudential.

Published Monthly.

M. B. HUTCHINS, M.D., BUSINESS MANAGER, No. 64 Marietta St.

### ORIGINAL COMMUNICATIONS.

### EXTRA-UTERINE PREGNANCY, WITH REPORT OF A CASE OF SIMULTANEOUS PREGNANCY IN BOTH TUBES.*

BY C. R. ROBINS, M.D., RICHMOND, VA.,
Instructor in Abdominal Surgery and Gynecology, and Visiting Obstetrician, Medical College of Virginia.

Extra-uterine pregnancy is a subject which demands the attention of every doctor who is responsible for the life and health of women. There is no woman, who may become pregnant, who is not liable to become the victim of this accident. It is at times so insidious in its development that its existence is not even suspected; and it may be so fatal that death may ensue before it is possible, with the greatest promptness, to render the necessary surgical aid. It undoubtedly occurs much more frequently than statistics can ever show, because, on the one hand, many cases recover without the trouble ever having been recognized, and, on the other, many cases

*Read before the Richmond Academy of Medicine and Surgery, Feb. 12, 1901.

die that are never brought to the care of the surgeon. This paper is a simple résumé of the subject, with the report of an interesting case.

*Varieties.*—While it has been claimed by eminent authorities that all cases of extra-uterine pregnancy are primarily tubal, this view is no longer held, and the following primary forms are generally admitted: (1) *Interstitial,* occurring in that portion of the tube which passes through the horn of the uterus. (2) *Tubal proper.* (3) *Tubo-ovarian,* occurring between the tube and ovary. (4) *Ovarian,* occurring in the Graafian follicle. (5) *Abdominal,* occurring in the abdominal cavity. By far the most common form, however, is that occurring in the tube proper; and our purposes tonight will be best served by discussing this, with a casual mention of the others.

*Pathology.*—When the fecundated ovum becomes implanted in the tube, the tube undergoes a process of thickening, which, however, is not due to a hyperplasia of the elements of the tube, but to a vascularization. The thickening is not uniform, but there occur certain thin spots, due to the stretching of the tissues, which mark the sight of future rupture.

In interstitial pregnancy rupture usually occurs into the free abdominal cavity. Rupture into the uterine cavity is possible, as is also rupture into and growth between the layers of the broad ligament. In tubo-ovarian pregnancy, rupture into the abdominal cavity usually takes place; but growth may occur inward between the layers of the broad ligament. It is possible for the fetus to develop to maturity. Ovarian pregnancy is exceedingly rare, and only a few cases have been reported. One of these went to term. Primary abdominal pregnancy is also exceedingly rare. The conditions favor the progress of pregnancy to term. Tubal abortion is a term applied to extrusion of the ovum through the abdominal end of the tube.

If a fetus survives these accidents, it may go on to term. In this case the woman will be taken with pains simulating normal parturition, accompanied by a discharge of blood and decidual shreds. This continues from a few hours to several days, when the pains cease and the tumor decreases in size, due to absorption of the fluids.

The hold which the ovum has on the walls of the tube is very precarious, and in a certain percentage of cases separation occurs, accompanied by hemorrhage resulting in the formation of a tubal mole. This mole may be sufficient to rupture the tube with the same results, which will be mentioned later. If the ovum continues to grow, rupture of the tube usually occurs primarily between eight and twelve weeks. If rupture occurs upward into the free abdominal cavity, it is usually associated with free and finally fatal hemorrhage. In cases, however, where the placental cite is downward, it is possible for the embryo alone to escape with only a moderate amount of hemorrhage; for a new sac to be formed from the peritoneal adhesions; and for the embryo to continue to derive its nourishment from the placenta attached to the walls of the tube. If death of the embryo occurs previous to two months, it is possible for it to become absorbed. It is possible also for hemorrhage to be limited by the formation of peritoneal adhesions.

If rupture occurs downward between the layers of the broad ligament, the hemorrhage is usually limited by the formation of a hematoma. If death of the embryo occurs, it is possible for the entire product to become absorbed. If the ovum continues to grow, subsequent rupture may occur, which may open up into the abdominal cavity. There is liability for secondary rupture and hemorrhage to occur even after the death of the embryo. After the second month the dead embryo cannot be absorbed and it undergoes either mummification or calcification, or decomposes.

At the time that these changes are taking place in the tube, the same changes that occur in normal pregnancy are taking place in the uterus—the formation of a decidual membrane and enlargement of the organ. The membrane becomes separated and is extruded between eight and twelve months usually, at the time of the death of embryo or rupture of the sac. It is from $\frac{3}{16}$ to $\frac{1}{4}$ of an inch in thickness, is not very friable, is extruded in large pieces or as a complete cast of the uterine cavity, and the uterine surface is covered with a thick, shaggy, villous coat which becomes apparent on being floated in water.

*Symptoms* should be divided into those occurring previous and subsequent to rupture.

(1) *Previous to rupture.*--This malady usually occurs in women who have never borne children, or who have not borne a child for a protracted period. There is also unusually a history of uterine disorder. Such a woman, having been menstruating with regularity, misses one or two periods. This is associated with the subjective signs of normal pregnancy. There is increase in the areola about the nipple; Montgomery's glands and the breasts become enlarged; gastric disturbances—anorexia, nausea and vomiting occur; there is the usual purplish tinge about the vagina; the cervix is softened and the os filled with a mucous plug. The woman often says she is pregnant. Associated with these normal changes, there is pain, which is characteristic, although in some cases it is absent. It may be dull and aching, but is usually sharp and lancinating, and occurs in paroxysms. It is described by the patient as being of the most violent character, and may commence at any time from a few days to several months after a normal menstruation. The paroxysms are separated by intervals free from suffering. The pain may be situated on one side, or may be indefinitely located in the lower abdomen. It may radiate down one leg or upward into the epigastrium. Sometimes it is so violent as to cause nausea, cold sweating, fainting and every evidence of severe shock. The temperature is almost always elevated, and may be quite high. The pain is usually accompanied by a return of a flow of blood, which may contain some shreds of decidua, and which continues irregularly for months. Instead of the usual amenorrhea, there may be irregular bleeding from the first, and in some cases there is a marked metrorrhagia.

(2) *Subsequent to rupture*—.Rupture is accompanied by characteristic symptoms. The patient is seized with an agonizing, lancinating pain in the abdomen. It may come on at any hour of the night, and is not dependent upon any undue cause. This pain is rapidly succeeded by signs of hemorrhage, which, if extensive, may cause the patient to fall unconscious to the floor; the pulse, at first rapid, becomes almost or quite imperceptible; the face loses all of its color; the body breaks out in a cold sweat; the extremities become cold, and the breathing sighing and finally jerky. Further symptoms depend on whether the bleeding continues or is limited. In the first instance, death follows in from a few hours to several days. If the rupture is confined within the tube or takes place between the layers of the broad ligament, or is confined by peritoneal adhesions, the patient gradually improves; but all of the symptoms may return or be intensified by a subsequent hemorrhage.

*Diagnosis.*—In a certain proportion of cases there are absolutely no indications until rupture occurs. In others the symptoms given in the foregoing occur with more or less distinctness. Those which should always excite suspicion are the characteristic pains associated with the signs of pregnancy, and irregular bleeding. Irregular bleeding, occurring in a woman who has previously menstruated regularly, should always excite our suspicions, even if not associated with other symptoms. When the characteristic pains occur, our suspicions should be thoroughly aroused.

Upon making a bimanual examination, the tube on the affected side is found enlarged and quite tender. If the enlargement is of sufficient size, the uterus is pushed to the other side. The tumor usually lies to one side of the uterus and curls around it posteriorly. A well-marked sulcus can be made out between it and the uterus. A sign that is mentioned, readily felt through the vagina, and which I have found well marked in two cases, is an accentuated pulsation in the ovarian artery of the affected side. After rupture, the diagnosis, from a consideration of the symptoms and bimanual examination, should be comparatively easy.

*Treatment.*—The treatment of extra-uterine pregnancy should invariably be surgical. When the diagnosis is made before rupture, operation consists of the removal of the affected parts by abdominal section. In a proportion of cases, where the symptoms are carefully considered, it is possible to make a diagnosis before rupture. In cases seen at the time of rupture, if the constitutional symptoms are not urgent, and there is a disposition for the patient to recuperate, and a well-defined mass can be made out in one or the other broad ligament, it is advisable to defer operation until it can be accomplished after due preparation, and the patient has sufficiently recovered to enable her to better withstand the shock.

Where rupture occurs in the broad ligament prior to the completion of the second month, some authorities advise against operating at all as the mass will probably be absorbed. The difficulty, in the first place, lies in ascertaining if the embryo is dead. Growth may continue with secondary rupture. Secondary and fatal rupture may take place after death of the fetus. Again, the woman will be left with a diseased appendage which will probably be the source of continued ill health. Suppuration may occur also. It is safer, in all cases, to operate.

The operation may be either vaginal or abdominal. The vaginal operation should be limited to those cases in which the rupture is undoubtedly intraligamentary and confined, and where the tumor is accessible to the vaginal finger for the complete romoval of clots and membrane. Even then it is open to the objection of the difficulty of securing satisfactory drainage, and the probability of leaving the patient, as mentioned before, with diseased appendages that will require a subsequent operation. Also, uncontrollable hemorrhage and other complications may arise, rendering abdominal section necessary after the already prolonged anesthesia and exhaustion of the patient.

Abdominal section must be undertaken in all cases where the bleeding is unlimited. The utmost expedition must be employed, as the delay of an hour may be fatal. In such cases the patient continues to grow worse, and examination reveals no defined tumor, but a bogginess may be felt in Douglas's *cul-de-sac*, due to the free blood in the abdominal cavity. After opening the cavity as rapidly as possible, every effort is directed toward securing the bleeding point and controlling hemorrhage. The hand is boldly plunged into the cavity, no attention being given to the accumulated blood, and the point of rupture is felt for and then compressed with the fingers until it can be secured with forceps. The blood and membranes are then removed, the cavity irrigated with hot saline solution, the affected parts ligated in the usual manner and removed, and the abdomen closed.

In those cases where the fetus goes to term, it is better to wait until its death before resorting to operation. Such a child is always poorly developed, and there is small chance of being able to prolong its life beyond a few days. There is, therefore, hardly any compensation for the additional risk to the mother of endeavoring to deliver a living child. After the death of the fetus, the placenta can be delivered with much less danger of hemorrhage or sepsis.

I wish to report the following case, it being, I believe, one of some rarity, and illustrating some of the conditions before described: I was called in consultation to see Mrs. X., who gave the following history: Age, 35 years; white; married; father died at about 33 years of consumption; mother living and healthy, aged 58 years; lost one brother by accident at 8 years. Her health, in childhood, was very good; she began menstruating at 16 years, from the beginning suffering very much for the first two days of each period, and usually spending the first day in bed. The periods were regular, rather free, and lasted about six days. She was married at 23, and has had three children, now aged 10, 8 and 4 years respectively. Last April she had a miscarriage at the sixth week, and since then has never been real strong, and has suffered somewhat with leucorrhea.

The last regular period occurred in the last part of July, missing in August. On September 9th there was marked nausea (which came on suddenly at 6 A.M.), attended by such great faintness and severe pain throughout the abdomen that she was compelled to lie down. On the next day she was attacked in the same manner while at the dinner table. On September 16th, at about six weeks' term, and that night, also, she passed some membrane and clots. This was unattended by pain, she being unaware of anything until she discovered the clots. Then followed considerable uterine bleeding, but she got up on the third day. Irregular bleeding continued until September 30th. On this day she ate very freely of watermelon and other fruit, and that night was taken with violent nausea, vomiting, and pain in the epigastrium and chest, and a stifling feeling in the throat. She became pallid and blind, and felt as though she were going to die. These symptoms were not relieved by vomiting, and continued throughout the night. Her physician thought she was suffering from an attack of acute indigestion.

The next day the pain and soreness were felt all through the lower abdomen. The irregular bleeding continued, the patient was very weak, and though the pain in the epigastrium had ceased, that in the lower abdomen was such as to prevent her from standing upright, and necessitated her remaining in bed four days.

I was not called to see the case until some weeks after the above occurrences, and my first examination was unsatisfactory, owing to extreme tenderness and rigidity of the abdominal muscles. I advised administration of an anesthetic, which was done several days later, when an enlarged tube, of the mass of a moderate size sweet potato, could be outlined on the left side, curling back behind the uterus. The case was diagnosed as one of extra-uterine pregnancy, which was confirmed by Dr. George Ben. Johnson, and operation was advised.

I operated on the patient on November 15, 1900. Section was made in the median line, and the enlarged tube on the left side, distended by blood-clot and bound down by adhesions, was revealed. The tube and ovary on this side were removed, and the other side explored. In Douglas's *cul-de-sac*, behind the right tube, there was discovered a round ball, about a half-inch in diameter and of a yellowish color, adherent to the peritoneum. The tube itself was in a thickened condition; and at about its central portion was a round perforation partially healed. The ovary on this side was in a condition of cystic degeneration. The tube and part of the ovary were removed, and the abdominal wound closed without drainage.

Examination of the left tube showed that rupture had not taken place; but the upper aspect was thinned to less than the thickness of tissue paper, and in the event of any subsequent hemorrhage, would surely have ruptured into the abdominal cavity. The distention was caused by blood which had become organized and undergone the changes due to the formation of a tubal mole.

Examination of the right tube showed that the perforation communicated with its interior, and the ball found in the free peritoneal cavity was a mole which had ruptured through the tube. It was not possible to determine the quantity of blood which accompanied this rupture as it, if any, had all been absorbed.

This was undoubtedly a case of simultaneous pregnancy in both tubes, with formation of tubal moles in both, and rupture of one tube. The woman made an uneventful recovery.

DISCUSSION.

*Dr. Jacob Michaux* said that the chief interest in this condition was the question of diagnosis; and he congratulated Dr. Robins upon the lucidity of his paper in this respect. So far as he was aware, diagnosing the condition before rupture was, in this city, uncommon. He had seen but one case, and at the time, bones were being passed by way of the rectum. The great difficulty in diagnosis consisted of the liability of confusing the condition with other maladies affecting the organs involved. The passage of shreds of membrane with blood, for instance, was liable to be mistaken for membranous dysmenorrhea, and it required a careful study of these fragments to make the diagnosis. In extra-uterine pregnancy they were much thicker and usually came away in small pieces, though sometimes as a whole. Another feature was that one woman might have a uterine miscarriage in which the fetus, unknowingly, and the membranes escaped; and another, before rupture, might have a mass on one or the other side, due to a variety of causes, *e. g.* pyosalpinx displacing the uterus or not, according to its size. The difficulty of making an exact diagnosis was extreme, and, under some conditions, impossible, and so when it was made, as in the case reported, the gentleman was to be congratulated.

The question of operation was chiefly interesting regarding the treatment of the placenta. Its removal, especially when attached outside, and to the walls of the abdomen and intestines, was a serious matter, and so much so, that the best writers advised its being left intact after cutting the cord close.

*Dr. George Ben. Johnston* said that for a long time he had been interested, in common with others doing abdominal work, in this subject, and he was convinced from his own observations and experience that a number of cases escaped detection and died from a curable malady. If practitioners were as faithful in the search for this trouble as for appendicitis, more cases would be met with, and those that had rupture treated successfully. He disagreed with Dr.

Michaux regarding the difficulty of diagnosis, for, besides the casting of membrane, there were other indications. It occurred after a skip or failure of menstruation in the sterile or one not pregnant for a long time. Very often the other signs of pregnancy were absent. Examination should be insisted on; it might reveal an unruptured tube, or an abnormal condition of tube or ovary. It would satisfy, and was not only necessary, but imperative. If the malady should turn out to be not extra-uterine pregnancy, but hydrosalpinx, pyosalpinx, etc., the tube could be removed. In any case, therefore, the matter was a surgical one; pain in the region of the pregnant tube, the condition of the cervix and vagina, and, finally, a distinct tumor, usually pulsating, aided in the diagnosis.

He was able to recall six cases in which diagnosis was made before rupture occurred, and five, including Dr. Robins's, were successfully operated upon. The sixth case was seen in consultation and operation was advised; but the physician in attendance agreed neither in the diagnosis nor advice. The case was submitted to the husband of the patient, who sided with the physician. Three days after he was called hastily, and found the patient in profound collapse and ready to expire. The family physician had arrived in the meantime, and a few minutes later the woman died with all the symptoms of a virulent, rapid hemorrhage. Post-mortem examination revealed a torrent of blood, the tube on the suspected side torn, and an escaped fetus.

Dr. Johnston thought that every case in which there was hemorrhage called for operation. Of course, those in which rupture had occurred into the abdominal cavity demanded promptest attention. These often died before the surgeon could reach them; but even those apparently hopeless should be operated upon, for the patient might not be dangerously exsanguinated. Where rupture occurred into the broad ligament, much might be done, the proper route being abdominal, not the vaginal. He had several times extracted clots through the vaginal opening only to be obliged later to go through the abdomen in order to stop a hemorrhage that could not be controlled otherwise. One case of peculiar interest that was diagnosed was hemorrhage into the broad ligament, followed by rupture of this into the abdominal cavity. He had operated in several cases in which hemorrhage had occurred into the broad ligament, the after-clot being large enough to occlude the opening. These cases did extremely well.

*Dr. J. W. Henson* reported the following case, seen in consultation: The patient at the time had been pregnant eleven or twelve months. At about the eighth month movement ceased, and soon after the size of the abdomen had begun to decrease. Ectopic pregnancy with death of the fetus was diagnosed. Upon opening the abdomen, it was found that rupture had occurred into the broad ligament, between the folds of which the embryo had developed, and that the head was in the floor of the pelvis, with the feet almost touching the liver. The sac was tense and almost ruptured from pressure of the feet. A good part of the sac was removed, the lower part, however, being allowed to remain as it was so vascular and investing all of the neighboring viscera, that any attempt at removal would have entailed dangerous hemorrhage. The remains of the sac were sutured to the peritoneum and a drainage-tube inserted. The abdominal wound was then closed except at its lower angle, where a second drainage-tube was inserted. The patient recovered.

